# A rare arteriovenous communication between left testicular vein with left accessory renal artery and multiple renal vascular anomalies: understanding the genetic and embryological perspective

**DOI:** 10.1590/1677-5449.202501252

**Published:** 2026-04-17

**Authors:** Sarah S. Sangma, Neerja Rani, Jessy JP, Rajesh Kumar

**Affiliations:** 1 All India Institute of Medical Sciences, Gorakhpur, Uttar Pradesh, India.; 2 All India Institute of Medical Sciences, New Delhi, India.; 3 All India Institute of Medical Sciences, Patna, Bihar, India.

**Keywords:** abnormal arteriovenous communications, varicocele, accessory renal veins, accessory renal artery, renal transplantations, comunicações arteriovenosas anormais, varicocele, veias renais acessórias, artéria renal acessória, transplantes renais

## Abstract

Vascular malformations are abnormalities of the vascular tree, either congenital or acquired, which may involve arteries, veins, capillaries, or lymphatic vessels. Congenital malformations, such as patent ductus arteriosus, may persist into adulthood, resulting in abnormal vascular communications. Acquired vascular communications may occur following trauma, such as gunshot or stab injuries, or after surgical procedures. Arteriovenous malformations are congenital disorders affecting the circulatory system during early developmental stages. Renal vascular variations are also relatively common, usually silent (asymptomatic) and often diagnosed incidentally during surgical or radiological procedures. Renal artery variations occur in approximately 30% of the population. Renal vein variations are also frequent but are always overlooked than their arterial counterpart. In this case study, we report an abnormal communication along with multiple urogenital vascular anomalies and discuss their genetic and developmental perspectives. Recognition of such anomalies helps establish safety guidelines for the diagnosis and management of renal and gonadal disorders.

## Resumo

Malformações vasculares são anomalias da árvore vascular que podem afetar artérias, veias, capilares ou vasos linfáticos, podendo ser congênitas e adquiridas. Malformações congênitas, como persistência do canal arterial, podem persistir em adultos, causando comunicações vasculares anormais. Comunicações adquiridas ocorrem após qualquer trauma, como ferimentos por arma de fogo ou facadas, e qualquer procedimento cirúrgico. Malformações arteriovenosas são doenças congênitas que afetam o sistema circulatório durante período inicial de desenvolvimento. Variações vasculares renais também não são incomuns e geralmente são silenciosas e assintomáticas, diagnosticadas incidentalmente durante procedimentos cirúrgicos e radiológicos. Variações da artéria renal ocorrem em 30% da população. Variações da veia renal também são bastante comuns, mas são sempre negligenciadas em comparação às suas contrapartes. Neste estudo, relatamos comunicação anormal e uma série de anomalias da vasculatura urogenital, sua perspectiva genética e de desenvolvimento. Tal anomalia fornecerá diretrizes de segurança para o diagnóstico e tratamento de distúrbios renais e gonadais.

## INTRODUCTION

Varicosities of the testicular veins within the scrotum and inguinal canal occur in approximately 15% of the male population. Varicocele is a well-recognized cause of male infertility, accounting for about 25-30% of cases. Varicocele formation occurs more commonly on the left side, which may be attributed to the orthogonal junction between the left testicular vein (LTV) and the left renal vein (LRV).^1^ An abnormal arteriovenous communication between the LTV and an accessory renal artery (ARA) may further contribute to the development of varicocele, as it results in shunting of blood from a high-pressure arterial system to a low-pressure venous system.^2^ To date, only two cases of abnormal arteriovenous communication between the testicular vein and testicular artery have been reported in the literature. Here, we report an extremely rare case of an arteriovenous communication between the LTV and the left ARA.

Renal vasculature variations are also common. Multiple renal veins have been reported in the literature but remain rare.^3^ The overall prevalence of renal vein variations is 24.7%, with multiple renal veins accounting for 16.7%.^4,5^

A single renal artery supplying each kidney is present in approximately 70% of individuals. ARAs are present in about 30% of individuals^6^ and are generally less than 2 mm in diameter. The reported prevalence in the literature ranges from 24% to 42%. Bilateral occurrence of ARAs is approximately 10%.^7^

Ethics statement/ethical approval: Ethical approval from the local institutional ethics committee was not required for this study, as the cadavers used were voluntarily donated. The authors also declare that the manuscript was prepared in accordance with the Declaration of Helsinki.

## CASE REPORT

**Figure 1:**
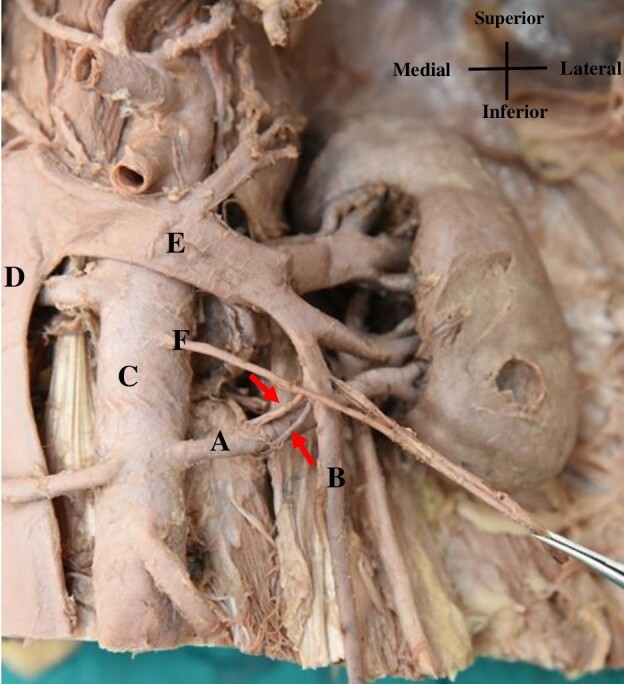
Image demonstrating an arteriovenous communication (red arrows) between (A) the left accessory renal artery and (B) the left testicular vein; (C) Abdominal aorta; (D) inferior vena cava; (E) left renal vein; and (F) left testicular artery.

During routine dissection of a 65-year-old male cadaver in the Department of Anatomy at the All India Institute of Medical Sciences, New Delhi, India, we observed a very rare communication between the LTV and the left ARA. Two communicating channels, each measuring approximately 0.5 cm, were observed connecting the two vessels, located about 2 inches from the origin of the left ARA and the termination of the LTV. The scrotum and testes were exposed and were observed to be normal (Figure 1).

Multiple bilateral variations in the urogenital vasculature were also observed in the same cadaver during routine dissection.

**Figure 2:**
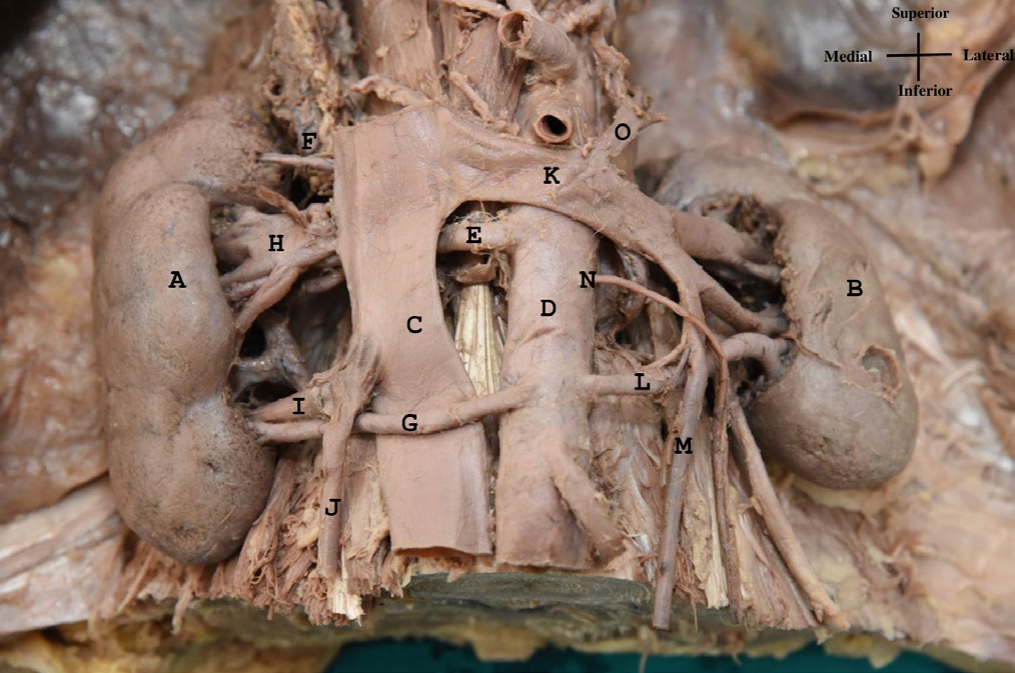
Image demonstrating multiple renal vascular anomalies. (A) Right kidney; (B) left kidney; (C) inferior vena cava; (D) abdominal aorta; (E) right main renal artery; (F) right upper accessory (polar) renal artery; (G) right lower accessory (polar) renal artery; (H) right renal vein; (I) right accessory renal vein; (J) right testicular vein; (K) left renal vein; (L) left accessory renal artery; (M) left testicular vein; (N) left testicular artery; (O) left suprarenal vein.

The right renal vein (RRV), measuring approximately 2.5 cm in length, was formed by the union of two to three segmental veins and drained into the inferior vena cava (IVC) at the level of the lower border of L1. An ARA was observed on the right side, draining the lower pole separately into the IVC at the level of L3. The right testicular vein (RTV) drained into the accessory RRV (Figure 2).

The LRV was observed to be longer (7.5 cm) than the RRV. It was formed by the union of two segmental veins 2.5 cm from the renal hilum and drained into the IVC at the level of L2. The left suprarenal vein was observed draining into the LRV. The LTV coursed superiorly and was found to drain into an inferior tributary of the LRV (Figure 2).

The right main renal artery was seen to originate as a lateral branch of the abdominal aorta at the level of the lower border of L1. Two ARAs were identified on right side. The upper ARA arose as a lateral branch from the abdominal aorta at the level of the upper border of L1. The lower ARA originated as a ventral branch from the abdominal aorta at the level of L3 and coursed anterior to the IVC to supply the lower pole of the kidney (Figure 2).

The left main renal artery was seen to originate as a lateral branch of the abdominal aorta at the level of the lower border of L1. One ARA was identified on the left side. It originated as a ventral branch of the abdominal aorta at the level of the lower border of L3 and coursed posterolaterally behind the LTV to supply the lower pole of the kidney (Figure 2).

## DISCUSSION

An abnormal arteriovenous communication is characterized by an atypical direct communication between an artery and a vein, thereby bypassing the high-resistance capillary bed. This results in shunting of blood from a high-pressure arterial system to a low-pressure venous system. Both arteries and veins develop from a common capillary plexus, which provides the developmental basis for the persistence of such direct arteriovenous communications.^8^

Recent molecular advances suggest that the ephrin family (EFNB2 and EPHB4) serve as markers for arterial and venous identity, respectively. Shear stress activates EFNB2 expression through vascular endothelial growth factor (VEGF)-NOTCH signaling, which governs arterial development and is regulated by hemodynamic forces. Downregulation of NOTCH signaling reduces the expression of arterial markers such as EFNB2 and NOTCH3, while increasing the expression of venous markers, including FLT4 and RTK5. VEGF also promotes arterial specification by downregulating Sonic hedgehog signaling and upregulating the NOTCH pathway.^9^

To date, only two cases reported in India have described an arteriovenous communication between the left testicular artery and the LTV.^10,11^ To our knowledge, the present case report is one of the rarest reported vascular variations and the first to describe an anomaly involving the gonadal vasculature in association with the left ARA. Such abnormal arteriovenous communication between the left ARA and the LTV may result in arterial blood into the venous system, thereby reducing arterial perfusion to the lower pole of the left kidney. This abnormal communication could also contribute to the development of a left-sided varicocele, potentially affecting testicular thermoregulation and impairing spermatogenesis. Furthermore, arteriovenous communications are often structurally weakened sites and may rupture, leading to hemorrhage and serious complications.

Compared with renal arteries, considerably fewer studies have examined variations in renal venous anatomy. Renal veins are formed by the union of segmental veins. Anjamrooz et.al. reported that the RRV was formed by the union of three segmental veins and drained into the IVC at the level of L1. The LRV was also formed by the union of three segmental veins and subsequently divided into superior and inferior tributaries. These tributaries were observed to drain separately into IVC at the levels of L2 and L3, respectively.^12^ These findings are comparable to those of the present study.

Our case is further distinguished by the formation of the LRV 2.5 cm away from the renal hilum, drainage of LTV into the inferior tributary of the LRV, and drainage of the RTV into an accessory RRV.

Hostiuc et al.^5^ described three major types of renal vein variants: (A) multiple renal veins or accessory veins, in which two or more renal veins drain separately into the IVC; this variant may be unilateral or bilateral; (B) the retroaortic left renal vein, in which the renal vein follows a retroaortic course before entering the IVC; and (C) the circumaortic left renal vein, which forms a venous ring around the aorta.

Accessory veins or multiple renal veins represent an anatomical variant that is more commonly observed on right side.^5,13^ A meta-analysis on multiple renal veins reported an overall prevalence of 16.7%, with a higher frequency on the right side (16.6%). The predominance of multiple renal veins on the right side has been attributed to differences in embryological development, as the complex embryogenesis on the left side prevents the persistence of additional veins. Multiple RRVs are formed by the persistence of embryonic renal veins arranged in a ladder-like pattern, whereas double LRVs result from the persistence of a central retroaortic venous anastomosis.^5,14^

The presence of multiple renal veins is considered a contraindication for donor nephrectomy, as it is associated with a higher risk of graft renal vein thrombosis. Consequently, the left kidney is generally preferred for laparoscopic donor nephrectomy because its longer renal vein facilitates vascular anastomosis during transplantation.^12^

Numerous variations in the venous drainage of testicular veins have been reported. Thakur et al.^3^ noted that anomalous termination of the testicular veins may contribute to the development and recurrence of varicocele. The origin and number of ARAs are complex and closely linked to renal embryogenesis. The lateral mesonephric arteries supply the mesonephros, metanephros, suprarenal glands, and gonads. These arteries consist of nine pairs, which are divided into three groups: cranial, middle and caudal. The middle group gives rise to the renal arteries, whereas the cranial and caudal groups give rise to ARAs.^6^ Several authors have classified ARAs using various terminologies, including aortic superior or inferior (polar), supernumerary, additional, supplementary arteries.^7^

Some authors have suggested that an unusual origin of ARAs may predispose to atheroma formation. Hemodynamic changes at arterial bifurcations can lead to reduced blood flow velocity; the resulting alterations in flow decrease shear stress, which in turn triggers vasomotor and inflammatory responses, ultimately promoting atheroma formation.^7^ Recipients of renal graft with ARAs or multiple renal arteries show a significantly higher incidence of vascular complications compared with those receiving grafts supplied by a single renal artery.^15^

In conclusion, the presence of abnormal arteriovenous communications and variations in the renal vasculature should be carefully evaluated preoperatively using Doppler ultrasonography and arteriography to ensure accurate diagnosis and optimal treatment planning.

## CONCLUSION

An abnormal arteriovenous communication acts as a parasitic circuit superimposed on the normal circulation, serving no physiological benefit but capable of producing deleterious effect such as elevated venous pressure and varicocele. According to basic principles of hemodynamics, blood (like water) flows along the path of least resistance, moving from a high resistance vessel to a low resistance vessel. Therefore, clinicians and surgeons involved in the management of varicocele and male infertility should be aware of such abnormal arteriovenous communications and confirm their presence using Doppler ultrasonography and arteriography.

## Data Availability

All data generated or analyzed are included in this article and/or in the supplemental material.
